# Lost in Context? Critical Perspectives on Individualization

**DOI:** 10.1007/s42087-022-00295-6

**Published:** 2022-06-15

**Authors:** J. L. Degen, A. Kleeberg-Niepage, P. M. Bal

**Affiliations:** 1 Department of Psychology, European University of Flensburg, Flensburg, Germany; 2 Responsible Management Research Group, Lincoln International Business School, University of Lincoln, Lincoln, UK

**Keywords:** Individualization, Neoliberalism, Critical psychology, Subjectivity

## Abstract

People in contemporary society are increasingly being addressed as agentic individuals who are held responsible for personal aspects of their life and beyond. These personal aspects contain the design and organization of one’s life path in terms of, e.g., (lifelong) education, work and retirement planning, health care, work-life balance, and happiness; or with regard to more abstract concepts like sustainability, individual subjects become responsible for the future of the ecosystem on a planetary scale. This individualization includes on the one hand potential empowerment of the subject to actively shape one’s own life, and on the other hand, it tends to ignore relevant socio-economic processes, scope, and power relations, which unfold as implicit and explicit social restrictions and potential pressure. Subjects navigate through such contexts with a compulsion to control faith and course of life by their decision-making, behavior, and an overall urge to optimize the self. This special section on individualization contains (a) an editorial frame of individualization within contemporary developments in a neoliberal context and (b) empirical contributions around the processes of individualization in various conditions such as the housing crisis in Berlin, career trajectories, and incorporated neoliberal ideology when opting out of a corporate career, pseudo individualization in Indian television commercials, and leisure activities alongside the example of soccer and related fan-group dynamics interpreted as an escape from the pressure to singularize.

## Some Thoughts on Individualization

Individualization has ambiguous meanings, being on the one hand the optimistic vision of a future grounded in values such as self-actualization, and personal freedom, and connected to such visions, self-centering as something positive to be achieved. On the other hand, such a constitution of the self is interpreted as the core of an expanding crisis blaming individuals for causing exploitation of each other and the ecosystem under an accelerating capitalist logic. However, it does not seem either-or, but complex negotiation processes of subjects confronted with a restrictive context. Evolving mechanisms in place reach into multiple, maybe all, spheres of life leading to the question: What kind of (social?) *subject* evolves under neoliberal-capitalist conditions of life?

Individualization once emerged under the promising idea of subjective self-actualization and freedom. Such ideals were e.g. manifested in the famous American Dream, where hard work shall lead to success accessible to anyone, and success symbolizes the extent of personal will and dedication. Fast-forward to today, where individualism comes with an altered meaning in the middle of a western world and an expanding neoliberal ideology. Far from the once optimistic vision, individualism now is viewed from a dystopian perspective, with public discourses calling out “hyper-individualism,” where prioritizing the self is primarily condemned as egoistic and harmful to both the collective and the ecosystem. Individualism then really bears a paradox, being a praised value and something to achieve, while at the same time being pigeonholed as the source of an impending crisis.

Within neoliberal ideology, subjects are “obliged to be free” (Rose, 1989, p.220), singularized and stripped from their actual social and environmental surroundings, interdependence, and overall conditions of life (Tironi et al., 2022). However, this does not come in the form of suppression of the subject by explicit restrictions of scope, but instead, it is effective by the subjects’ internalization of the functions of the regulation (Rose, 1989).

Neoliberal ideology puts subjects subtly in responsibility for shaping and standing trial for *their own path of life*, meaning-making, and happiness—one is no longer a victim of class membership, social belonging, or dispositions. Such responsibility and pressure are met by (collectively reproduced and internalized) subjective optimization and strategies within the own scope, including decision-making, (lifelong) education, work, retirement planning, health management, and work-life balance (Gergen, 2006, 2014; Rose, 1989). Formerly, these strategies were principally based on dedication and hard work; nowadays, they are changing in quality and into a logic of outsmarting the system. This is because subjects are not only held responsible for their own success and faith but moreover to save time and effort, which again is constituted as the subjects’ number one scant resource (Rosa, 2015). The appeal then lies in the provision of a shortcut to success, where one is no longer determined by the own professional potential. Instead, if in possession of some general intelligence and common sense, one is supposedly able to achieve societal success and status by outsmarting work and exhaustion. This means that former understandings of work, including (timely) commitment and effort, turn into an aim for pseudo work—a way of tricking work—and are shown, e.g., by increasing stock trading and cryptocurrency investment.

On more abstract levels, subjects become responsible *for the future of the ecosystem on a planetary scale*, e.g., by lifestyle and consumption choices (Morton, 2016) or to cope with worldwide phenomena like the current pandemic by individual action and agency (Picione et al., 2021). At the same time, values such as sustainability, diversity, inclusion, and social justice are perceived through neoliberal (and capitalist) viewpoints, as means to an economic end in the marketization of democracy and a coaptation of humanist values (Gandesha, 2018). Through economic adaptation, such values then neatly fit within the contemporary form of neoliberal ideology, leading to a form of woke-capitalism (Rhodes, 2022). Within this dynamic, social and contextual (economic, ecological, and cultural) struggle is individualized and transformed into something that should be solved individually. While individual action (e.g., recycling, becoming vegan) might be a necessary step toward societal transformation, it often remains within the constraints of individual action and can be easily co-opted through a neoliberal logic (e.g., living vegan is a healthier lifestyle through which one can be a more productive citizen and a big-spending consumer). This shows the complexity of escaping one’s predicament, and the perpetual hijacking taking place within the contours of contemporary society, whereby individualization becomes self-referential: when one is profoundly unhappy with the individualized state of being, one is individualized to fix oneself and offered more individualization. However, the ongoing discussions set aside, success remains unequivocally and continuously measured in and pursued as individual wealth, which beyond existential security, and the nations’ wealth is knowingly neither meaningful in the long term nor grants happiness or savor (Ma & Zhang, 2014; Quoidbach et al., 2010).

What remains for the individual is to constitute the self by market and investment choices reinterpreted as agency (Besley & Peters, 2007). Such agency (through consumption) again is comprehended as an emphasis on difference, where taking care of the self and speciesism are prioritized over the other (including other species and the ecosystem), while loyalty, commitment, coexistence, and responsibility for each other are exchanged for self-realization. Hence, individualization seems to be sold under a neoliberal concept that really is self-centered consumerism, containing little of the former optimistic vision, but evidently leading to alienation and loneliness and an overall repulsion of positive individual capabilities in a world perceived as unsocial, competitive, and ruthless. Consequently, the collective becomes a mere collection of a non-integrative mass of subjects urging for individualization, seemingly existing in isolation and detached from the other (Elias, 2001). Overall, this sketches a state of being that is questioning the construction of a social self as such (Gergen, 2006, 2014).

Individualization then entails being lost in context, as social contexts no longer explicitly structure life but remain only at the margins as an invisible structure pushing for individualization. Much like the famous neoliberal axiom, there is no such thing as society, indicating the breakdown of society as such, and more importantly, the disappearance of a notion of the social context including guidance and security being brought down to oneself, and what is directly surrounding (Bettache & Chiu, 2019).

In sum, the contemporary individual finds oneself in a society in which one is not only responsible for the self, being stripped of societal protection and benefits (Marvakis, 2019), but where collectivity has slowly evaporated as a concept. Nevertheless, under the latest exaggerated and polarizing conditions of COVID-19 regulations and politics, structural inequalities and unequal effects on subjects push back on ideas of the subject being the agent of their own faith and success, supposedly independent of conditions in life. This is exemplarily shown by research on the pandemic, demonstrating the crucial role of context by showing that it is (again) the underprivileged who are most of all negatively impacted under pressure: e.g., the rise of gender inequality (Cameron et al., 2021), increasing and returning social injustice (Jović, 2021), and the negative effects on children and education depending on their background (Szulevicz, 2021).

## The Special Section: Evidence on Individualization in the Arena of Subjectivity

A special section on individualization signifies a search for the ‘invisible’, that “*what*” that has been internalized into the core fabric of society to such an extent that it is no longer visible but taken for granted and accepted as is. Putting the spotlight on the constitution of an individualized society highlights the uncomfortable nature of contemporary society for the modern citizen and the complex nature of the social context in which individuals have to enact within their allocated agency, notwithstanding their capabilities or chances to do so. While societal structures seem as determining as ever, the difference from previous eras may be that such structures are no longer admitted as such.

Such individualization of society has various implications for the understanding of the individual subject—a concept yet to be comprehensively defined in psychology—just as broader issues, such as organizing in society and including severe consequences for the subjects. What does it mean to live in an individualized society—especially with its 50-year “celebration” of neoliberal policy (starting in the 1970s), amplifying individualized neoliberal capitalism?

This Special Section on Individualization *“Lost in Context” in the Arena of Subjectivity* is aptly titled, as the contributions reflect the wide variety through which contemporary global experiences of individualization manifest; not just as a “psychological experience” of self-reliance and individual responsibility for one’s life but an all-encompassing now—the entirety of the special section shows how precarious social life has become, touching various aspects of life.

We explicitly encouraged innovative contributions in line with the principles of the hosting journal, Human Arenas, and its editor’s critical stance and commitment to change pushing the normative boundaries and conventional research approaches (Tateo & Marsico, 2022). We are pleased to present papers addressing various contexts, where individualization manifests through a variety of ways penetrating everyday life: through advertising and globalization, finding a home and housing, work-life and life-course under the incorporation of neoliberal ideology, and leisure when attending soccer games.

Roy and Putatunda (2022) discuss individualization as being far from a “Western project”—it is globalized jointly with capitalism: e.g., in India where it is observed that in advertising, traditional lifestyles merge smoothly with individualist consumerism, instrumentally using social debate (on gender equality and feminism) to further capitalist goals.

Reichertz (2022) addresses individualization and singularity as a phenomenon one has and wants to escape from. Following his argument, this escape forms through the collective bond found in soccer fan behavior. However, far from being innocent, this is an escape from individualization that has totalitarian potential, with a willingness to submerge oneself into violent action, a process whereby identification with one’s group members, one’s clan, can lead to violent clashes with “the Other.”

Tommasi and Degen (2022) analyze individualization in career trajectories and decision-making, manifesting as incorporated neoliberal ideology remaining in subjects even when opting out from corporate careers and pursuing alternative life and work styles and “keeping a foot in the door.”

Wolf (2022) presents an analysis of subjects in the process of finding a home, where unsocial competitive market conditions of life are negotiated within the self and the solution of finding an affordable home is interpreted as an act of performance, where the disadvantaged subject has to try as hard as one can to overcome the conditions of the market.

The articles included in the special section contribute to exploring the constitution of the social self by detailed analysis scrutinizing the tension between subject, agency, and restrictive contexts including respective principles, logic, and mechanisms. Such narrow confrontation sheds light on how subjective strategies are developed to meet the conditions in everyday life under an ever-expanding neoliberal capitalist logic. The contributions focus on varying themes (leisure, work, media, existential needs) showing how interwoven all spheres of life are with a neoliberal-capitalist logic, how relentless such contextualization manifests within the subjects, and how subjects most often react by incorporating such conditions, finding agency and dignity solely in their individual behavior and strategies. Understanding such micro-processes alongside concrete examples may finally lead to—in a critical psychological understanding of the potential for change through enlightenment—broadening the general subjects’ scope. In line with Gergen, we trust that a scholarly reconstruction of the self and its context will contribute to shaping a future by developing reflected, conceptual practices, which may “bring the alienated into forms of mutual coordination” (Gergen, 2006, p. 123).
